# Opportunistic plant observations reveal spatial and temporal gradients in phenology

**DOI:** 10.1038/s44185-024-00037-7

**Published:** 2024-03-06

**Authors:** Michael Rzanny, Patrick Mäder, Hans Christian Wittich, David Boho, Jana Wäldchen

**Affiliations:** 1 Department Biogeochemical Integration, Max Planck Institute for Biogeochemistry, Jena, Germany; 2 Data-Intensive Systems and Visualisation, Technische Universität Ilmenau, Ilmenau, Germany; 3 Faculty of Biological Sciences, Friedrich Schiller University, Jena, Germany; 4 iDiv, Leipzig, Germany

**Keywords:** Plant ecology, Phenology

## Abstract

Opportunistic plant records provide a rapidly growing source of spatiotemporal plant observation data. Here, we used such data to explore the question whether they can be used to detect changes in species phenologies. Examining 19 herbaceous and one woody plant species in two consecutive years across Europe, we observed significant shifts in their flowering phenology, being more pronounced for spring-flowering species (6-17 days) compared to summer-flowering species (1-6 days). Moreover, we show that these data are suitable to model large-scale relationships such as “Hopkins’ bioclimatic law” which quantifies the phenological delay with increasing elevation, latitude, and longitude. Here, we observe spatial shifts, ranging from –5 to 50 days per 1000 m elevation to latitudinal shifts ranging from –1 to 4 days per degree northwards, and longitudinal shifts ranging from –1 to 1 day per degree eastwards, depending on the species. Our findings show that the increasing volume of purely opportunistic plant observation data already provides reliable phenological information, and therewith can be used to support global, high-resolution phenology monitoring in the face of ongoing climate change.

## Introduction

Phenology, the timing of season-related life cycle events, plays a key role for plants and influences major processes such as growth, reproduction and evolution. Global warming and the related altered temperature and precipitation regimes^1,2^ but also urbanisation^3^ and deposition of CO_2_ and nitrogen^4^ are affecting the timing of plants’ phenological phases. In turn, altered plant phenology feeds back on global ecosystems and influences fundamental processes such as the carbon and water cycle^1,5,6^, ecological interactions^7–13^, or land-atmosphere interactions^14,15^. Especially, temperate vegetation is sensitive to climate variability, since temperature is a core driver of phenological changes in these regions^16,17^. These co-dependencies highlight the crucial role of phenology and the vital importance to adequately document, monitor and model changes in the timing of phenological events on larger scales and at more fine-grained resolution. However, current phenology monitoring is diverse in terms of scales, geographical regions, and approaches^18–20^. At the same time, there is an urgent need for phenology data suitable to parameterize predictive phenology models^20,21^ and to analyze effects on community level^18,22,23^. Therefore, phenological observations need to be integrated and extended with a focus on a broader taxonomical scope, especially towards herbaceous species, a higher spatial and temporal resolution, and a larger spatial extend^24,25^.

The digital age with almost ubiquitously available and high-performance technology makes way for a new type of data that can be harnessed for ecological research^26–29^: opportunistic plant records, collected via species reporting platforms, e.g., iNaturalist^30^, observation.org or artportalen^31^; and via identification apps, e.g., Flora Incognita^32^ and Pl@ntNet^33^. These plant observations have not been intentionally generated to address phenological questions, but come in large quantities, broad spatial coverage, and at high spatial, temporal and taxonomic resolution. Observations captured by these platforms already surpass the number of manually recorded phenology observations by orders of magnitude and unlock a great potential to study spatially and temporally highly resolved changes in species observation patterns^34^.

Phenology is known to primarily respond to temperature and precipitation. Therefore, it is dependent on geographic factors such as elevation, latitude and longitude. In the Northern hemisphere, spring events are observed later at higher latitudes, whereas autumn events occur earlier^35^. The same applies for higher elevations, where the average temperature decreases as altitude increases. These relationships have been described as the Bioclimatic Law, which hypothesizes that on the northern Hemisphere, phenological events shift by four days for one degree latitude north, five degrees longitude west, or 400 ft (120 m) of elevation increase^36^. Similar responses can be expected for the southern Hemisphere, although much less studied^37,38^. While this law neglects important sources of variation, such as differences between species, populations or regions^39–42^, its predictions have been described as matching observations derived from remote sensing, phenocams or ground observations^41,43,44^ when applied at coarse-grained scales. A recent study found that the Bioclimatic Law was systematically altered by global-change-induced warming for four tree species in the Swiss alps^43^. Trends predict consistently advancing phenological events of two to ten days per decade, depending on observed species and bioregion^1,35,45^.

In this study, we assess the viability of using large amounts of opportunistic plant observations to monitor changes in phenological patterns in 20 mostly herbaceous species with high temporal and spatial resolution. To achieve this, we calculate the median flowering dates for 20 species and compare the results across two years (2020 and 2021) on a grid across Europe. Until now, large-scale bioclimatic relationships have mainly been studied for tree species through dedicated local phenology observing initiatives. Our goal is to showcase how massive numbers of opportunistic plant observations can be used to quantify similar relationships for herbaceous species on a continental scale.

## Results

**Fig. 1:**
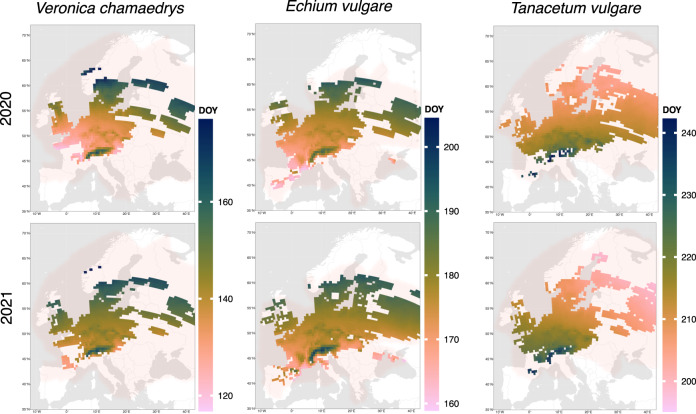
Median observation date (MOD) for *Veronica chamaedrys*, *Echium vulgare*, and *Tanacetum vulgare* in 2020 and 2021, estimated from opportunistic plant observations. MOD was calculated for grid cells of 50 × 50 km size with at least 20 observations. The day of year (DOY) designationg MOD is colour coded. Cells without sufficient observations are interpolated using a support vector machine (SVM) model based on elevation, latitude and longitude that was trained on the gridcells with sufficient observations. We interpolated only grid cells within the area of applicability, i.e. gridcells whose parameter can reliably estimated based on the observed MODs. The underlying red-shaded area visualizes a species' range of distribution. Note that the color scales differ between species.

We studied the flowering phenology of 20 species on a 50 × 50 km grid cell raster across Europe in terms of median observation date (MOD) per species and gridcell. In total, our analyses are based on 2,040,418 opportunistic plant observations. Figure 1 exemplary visualizes the median observation date per grid cell for the spring-flowering *Veronica chamaedrys*, the spring-to-summer flowering *Echium vulgare*, and the summer-flowering *Tanacetum vulgare* in 2020 and 2021. The maps for the remaining species can be found in SI Fig S6. In general, the three exemplary species show spatially varying phenology dependent on latitude, longitude and elevation (e.g., the European Alps), a considerable shift among the two years of observation, and a reversed shift in flowering between the early and the late-flowering species. The figure shows the species’ natural distribution range as a red-shaded overlay, verifying that the utilized observation data covers a substantial part of it.

### Species’ phenology within the growing season

**Fig. 2:**
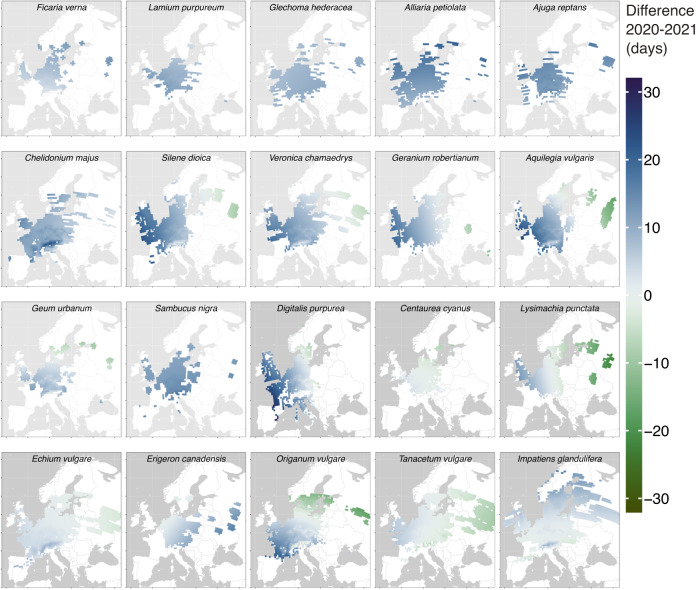
Differences in median observation date (MOD) for each studied species and per analyzed grid cell between 2020 and 2021. Blue colours indicate an earlier median flowering date in 2020 while green colors indicate an earlier median flowering date in 2021.

Figure 2 provides a spatially more detailed overview of the differences discussed above. Each map in the figure represents one species and shows differences in median observation date per grid cell between the two years. The maps are ordered according their median MOD in 2020. Although the extend of this time shift differed between species, there is a tendency towards a larger shift (6–17 days) for the spring-flowering species, whose mean date of observation was between mid April and mid June (DOY: 94–172). This shift levels off to a few days (1–6 days) in the course of the growing season until the mid of August (SI Figure S4). While almost all medians of MOD were observed earlier in 2020 than in 2021, there are local differences between species. For example, the median observation date for early-flowering species, such as *Ficaria verna, Lamium purpureum, Glechoma hederacea, Alliaria petiolata, Ajuga reptans*, and *Chelidonium majus* was advanced in 2020 in all observed regions, the median flowering date of later-flowering species was delayed in 2020 compared to 2021 in the eastern *Silene dioica, Aquilegia vulgaris, Tanacetum vulgare* and northern *Origanum vulgare* parts of Europe.

### Yearly temperature differences influence phenology

**Fig. 3:**
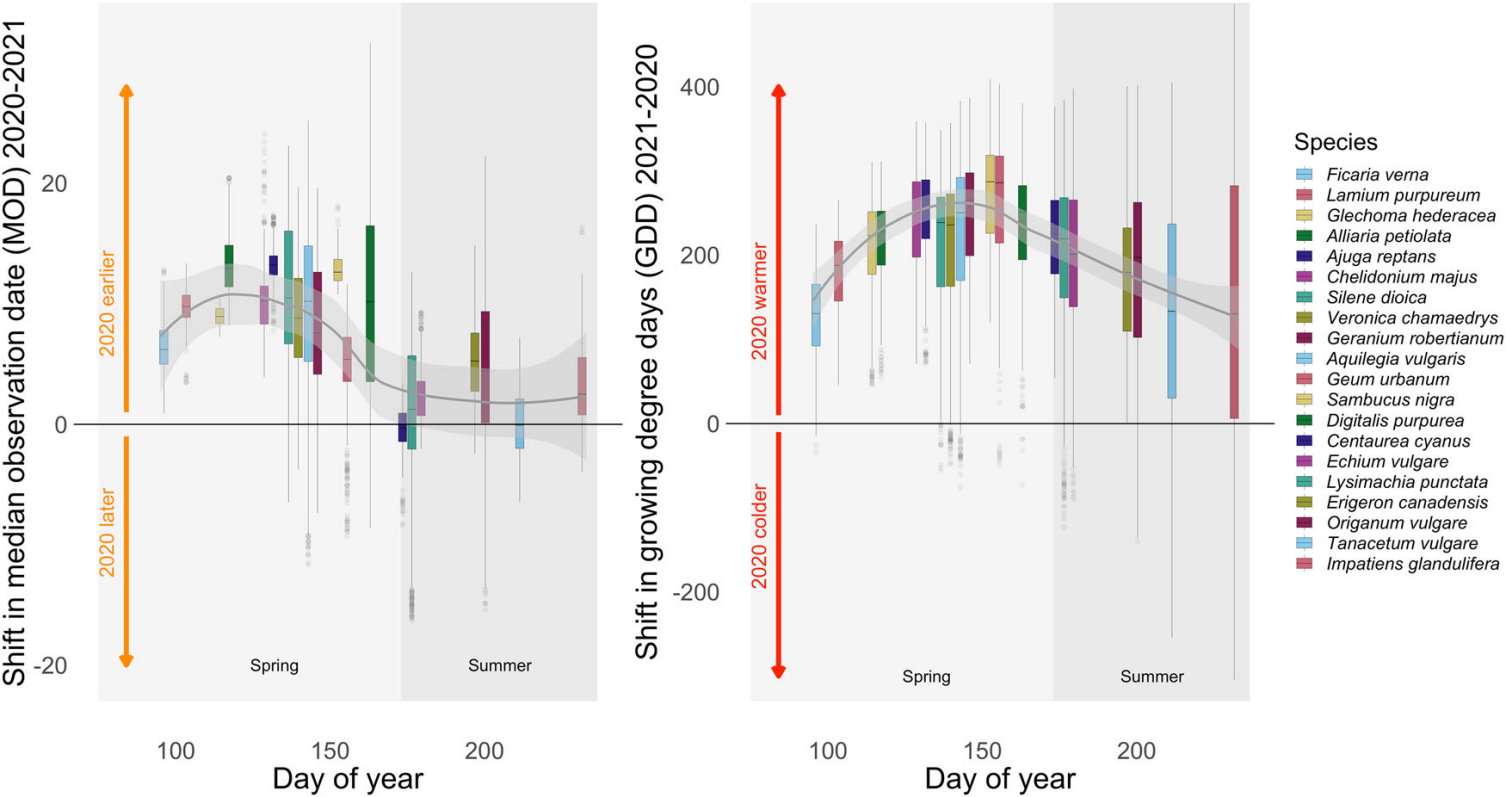
Shift in median flowering date (MOD) in relation to the shift in growing degree days (GDD). The boxplots on the left panel represent the difference in MOD for each species. Each boxplot shows the distribution of shifts in MOD across grid cells. The species on the x-axis are arranged according to their median MOD in 2020. The boxplots on the right panel show the difference in shift of GDD 2021-2020 across the same grid cells at the time when MOD of a particular species occurred in these grid cells. The x-values of the boxplots are identical in both panels. The grey line represents a smoothing function through the medians of each boxplot with the associated 95 % confidence interval indicated as a grey shade. For better comparability, we inverted the difference between 2020 and 2021 (i.e. 2021-2020 instead of 2021-2020) for the shift in GDD, as the relationship between both shifts is complementary. If a species is flowering earlier in 2020 than in 2021, the difference 2021–2020 would be positive. For the shift in GDD the expectation would than be that the accumulated temperature at the time of median flowering would be higher in 2020 than in 2021, resulting in a negative difference. Each species is color-coded in the same way across both panels. Values above zero on the left panel (upper orange arrow) indicate species observed earlier in 2020 than in 2021, while positive values on the right panel (upper red arrow) indicate that the accumulated temperatures (GDD) were higher in the respective grid cells in 2020 than in 2021. Negative values imply the opposite in both panels, i.e., MOD occurring later in 2020 compared to 2021 and GDD being lower in 2020 compared to 2021.

When studying the spatially aggregated medians of MOD per species and year, we found that all but one species (*Centaurea cyanus*) was observed significantly earlier in 2020 than in 2021 SI Table S1. In order to better understand and validate these inter-annual changes in the phenology we compared the median shift in MOD with the median shift in growing degree days (GDD). The left hand side panel of Fig. 3 shows the median shift of MOD across all grid cells as box plot per species. The panel on the right-hand side shows the difference in growing degree days (GDD) between 2020 and 2021 for the same gridcells at the time when median flowering date was observed for a particular species in these gridcells. We observed that the earlier flowering in 2020 is matched by a similar shift in GDD towards faster temperature accumulation.

### Bioclimatic variables influence phenology

**Fig. 4:**
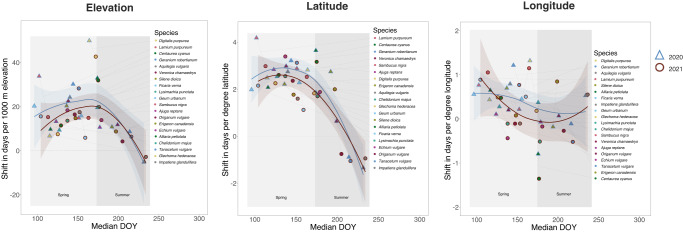
Relationship between shift in elevation (left), latitude (center), and longitude (right); and the median observation date per species in 2020 (triangles with blue margins) and 2021 (dots with red margins). The filling color of the symbols refers to the particular species. Median DOY (day of year) on the x-axis refers to the median observation date (MOD) across all gridcells in 2020 (blue dots) and 2021 (red dots). The species are color-coded and the symbols of the same species are connected with a dotted line. Curves are fitted using a LOESS smoother with span=1.5 and the shaded area represents the associated 95% confidence intervals. The background grey scales refer to spring -flowering (light grey) and summer-flowering (dark grey).

Figure 4 visualizes the relationship between the shift of a species’ median MOD in 2020 and 2021 for these three spatial dimensions; and the average shift in this period per 1000 m elevation, one-degree latitude and one-degree longitude. In general, flowering was belated towards increasing elevations and towards higher latitudes, except for very late-flowering species, while longitude was observed to have a more species-dependent effect if at all. In terms of elevation, the strongest influence was observed for *Digitalis purpurea* in 2020, showing a delay of 49.92 days in MOD for every 1000 m increase. Plotting the shift in days per 1000 m increase in elevation for all species against MOD results in a hump-shaped relationship, with its maximum during the transition from spring to summer in both observed years. The largest influence of latitude was observed for *Lamium purpureum* in 2020, with a delay of 4.18 days in MOD per degree northward occurrence. We observe an average delay of 2.11 days per degree northwards occurrence across both years for all species, while on the extreme, the three late-flowering species *Origanum vulgare*, *Tanacetum vulgare*, and *Impatiens glandulifera* flowered 0.75 to 1.05 days earlier per one degree northwards in 2021. The functional relationship across all species once again shows a hump-shaped pattern, peaking in the middle of spring, with the values for 2020 slightly above those for 2021. Regarding longitude, the most significant influence was observed for *Digitalis purpurea* in 2020, showing a delay of 1.32 days in flowering for every one degree eastwards. Similarly, *Centaurea cyanus* in 2021 exhibited earlier flowering by 1.35 days for every one-degree eastward occurrence. The relationship between longitude and median MOD are less consistent. For several cases, longitude has no significant effect in the statistical model.

## Discussion

In combining data from species-reporting platforms and identification apps, we were able to compile a comprehensive multi-species dataset allowing us to simultaneously compare the phenology of 20 plant species across two subsequent years and locations. Our study shows that opportunistically collected plant observations capture changes in the phenology of these species and can provide detailed information on large-scale phenological patterns along broad geographic gradients. This finding paves the way for a variety of further studies regarding changes in bioclimatic patterns not assessable before. Since the number of available observation records is expected to further increase^26^, this data can provide highly relevant large-scale information not limited by the borders of federal states, which may or may not monitor the phenology of species, but rather by the occurrence of the focal species. At the same time, analyses at highly spatially, taxonomically, and temporally resolved levels become possible. Due to limited data availability, earlier studies using opportunistic data had to use records aggregated from several years^23,28^, thereby necessarily neglecting the considerable inter-annual variability, which can be as large as two weeks for some species, as we have shown in this study. Additionally, very sparse observation counts can result in inaccurate predictions and misleading conclusions^46^, especially if less robust estimates, such as onset of flowering, are estimated from that data. Being based on more than two million entirely opportunistic plant observations and using MOD, a more robust phenometric suitable for opportunistic observations, our work shows that these data provide a means to continuously monitor wildflower phenology on an annual basis.

Plants respond differently to climate change. While some species may respond with, e.g., earlier flowering, other species show no trend at all^27,47–49^. Different aspects of temperature alone^50^ do not explain phenological variation of various temperate plants under experimental warming^51^. Other factors, such as the number of chilling days or the length of the photo period per day are known to affect the phenology of species, too^27,41,52^. On larger scales, however, accumulating growing degree days with the standard base temperature of five degrees Celsius represents a simple, yet accurate model for predicting the flowering phenology of plants^53,54^. Summer-flowering species have been reported to show more inter-annual variation than spring-flowering species, while the latter are more strongly correlated to mean monthly temperatures^55^. Early-flowering species require larger time spans to accommodate for the smaller accumulated forcing temperatures early in the vegetation season^56^. The results from our showcase covering two years are in line with those findings. In most parts of Europe, the accumulated temperatures in 2021 were lower than in 2020. The spring-flowering species responded with a larger shift than the summer-flowering species (SI Table S1). In contrast to the strong inter-annual differences in the DOY of median observation date, the same species show much less inter-annual variation in the GDDs accumulated upon their mean flowering dates (SI Fig. S8).

Elevation- and latitude-induced shifts have previously been quantified mostly for leaf-out of woody plants^36,41,43,57,58^. Based on dedicated phenological observations in the Swiss Alps, Vitasse et al.^43^ found that Hopkins’ bioclimatic law of spring phenology has changed since originally formulated in 1920. The elevation-induced phenological shift of the tree-leaf-out date decreased by 35% from 34 days per 1,000 m in 1960 to 22 days per 1,000 m in 2016. Although the absolute values differed between the four observed species, all showed the general trend of decreased elevation-induced shift. Another estimated delay for leaf-unfolding in beech trees is reported of being 26 days per 1,000 m increase in elevation^57^. Our results show even more decreased shifts for several of the earliest-flowering species, occurring around the time of tree leaf-out (DOY 100-130). Averaging the values of the six species whose mean observation dates fall into this range results in a mean of 15.5 days in 2020 and 13.6 days in 2021, with values ranging from 6.7 days (*Glechoma hederacea*, 2020) to 33.8 days (*Lamium purpureum*, 2020) (cp. SI Tab S2). This elevation-induced shift shows a hump-shaped relationship over the course of the vegetation season (cp. Fig. 4 left). This indicates that for later-flowering species, the trend of belated flowering towards higher elevation and latitude is weakened and ultimately reversed. Populations of *Impatiens glandulifera* that stem from higher latitudes have been shown to produce flowers earlier than populations from lower latitudes^59^. *Impatiens glandulifera* is an invasive species in Europe and is, among others, limited by temperature^60^. As the length of the vegetation season is shorter at high elevations and latitudes, the earlier median observation dates in our study are in line with these findings. Hopkins reported a four-day shift in spring phenology per degree northward and five degrees westward for the US in 1960^36^. In our study, the mean estimates for early-flowering species considered for elevation are 2.7 days in both years, which is reasonable considering the observed decrease in elevation-induced shift. A more recent study reported a delay of 2.7 days per degree northwards and 21 days per 1,000 m elevation for the greening in deciduous forests based on phenocam data^41^. However, the authors of that study did not find similar relationships for the greening of other observed ecosystems (evergreen needle leaf forest and grassland vegetation). Similar to the elevation-induced shift, the latitude-induced shift shows a maximum value around DOY 150 as a consequence of the shorter vegetation period. Only some of the observed species show a significant response along the longitude component in our regression models. While latitudinal and elevational gradients represent fundamental temperature gradients in temperate climates, the relationship in longitudinal direction is not that strong and might differ across regions. The North American land mass, for which Hopkins developed his bioclimatic law, is likely to show different gradients than the Central European land mass, e.g., with respect to climatic continentality.

The large numbers of available community observations also comes with associated problems. In the following we briefly discuss the assumptions we made and the potential impact of biases that are inherent to opportunistic plant observations and to citizen science data in general. These biases include, e.g., weekend effects, holiday effects, weather effects or changes in user motivation^61^. While fully accounting for this mix of potentially interdependent biases is not possible, we took some precautionary measures to mitigate their impact. Most importantly, an evaluation against independent data from dedicated monitoring efforts can provide information on the reliability of the data. Katal et al.^62^ explored how plant observations, collected via a plant identification app, can be compared to data collected by trained phenology observers and found largely corresponding patterns in the onset of flowering estimates for species with a conspicuous flowering stage. This indicates, on the one hand, a potentially strong link between flowering phenology and local observation density, and, on the other hand, a tolerable impact of the underlying biases on the estimated phenology measure. In contrast to the latter study, we decided to analyze the median observation dates, i.e., the 50th percentiles of a Weibull function fitted on the distribution of observations dates in each gridcell. This approach has been shown to represent a most robust phenometric for opportunistic data^63^. These authors argue that they achieved unbiased estimates for as few as ten individuals in some cases^63^. In order to increase the robustness of this approach we required at least 25 observations per gridcell to calculate their MOD. The number of observations per gridcell can vary considerably, as opportunistic observations are often locally clustered in highly populated areas^34^. The probability of recording extreme data points increases with the number of observations, which has been shown to affect the estimation of the day of onset of flowering, but much less the day of median observation^64,65^. Therefore, we favored ‘median observation dates’ over ‘first appearance dates’ in this analysis, as median dates have been shown to be more accurate and less sensitive to sampling than the onset of flowering, especially when using partially sparse plant observations without known phenological stage^8,9,66–68^.

We used opportunistic observations from 6 different sources. Flora Incognita and Pl@nt net represent plant identification apps and contribute almost always considerably more than half of the observations per species (see SI Fig. S2). The common observation patterns become already apparent from the overall distribution. The systematic shift of the peak within Swedish Artportalen and the Norwegian NCBI represents a phenological consequence, as observations contributed via theses platforms were collected in Northern Europe, where mean temperatures are lower than in Central Europe, where the main fraction of observations from the other data sources were collected (see SI Fig. S4). However, as the number of observations from the Nordic sources are very sparse for some species, the density curves are strongly influenced by single observations. Another source of error might be that opportunistically collected images do not carry information on the exact phenological phase of the plant. However, in order to compare large-scale patterns, it is not necessary to define the concrete pheno phase. We assume that user behavior in documenting plant species is similar for the same species across regions and years for large observation numbers. The density curves in Fig. S2 imply that this is a reasonable assumption. Our approach requires aggregating species’ observations across larger spatial units, whereas dedicated phenological observations are usually collected on individual plants. Hence, our derived phenometric estimate represents the community average of a series of unstructured presence-only records within the chosen spatial unit, not individual records. In consequence, small-scale phenological variability is aggregated on a larger scale, while the sampling effort is not uniform across the considered spatial units^64^. Therefore, our current approach will inevitably be biased towards densely populated areas as here the number of observations is higher (see SI Fig. S4)^34^. In some gridcells, thousands of observations were available for some species per year, which would allow for a much higher resolution locally, given that 25 observations were necessary to estimate median observation date. There is a general trade-off between smaller gridcells and better-resolved estimates but more sparse coverage and larger gridcells with more aggregated estimates but better coverage. It remains an open question under which circumstances which approach is more suitable. While this could potentially influence the absolute phenometric estimates as, e.g., urbanization might affect phenology^3,27,69^, we assume the potential effect on the inter-annual differences to be negligible, as population and observation density are unlikely to shift systematically between years. This is underpinned by the highly consistent inter-annual shifts regardless of the observed species (Fig. 2). In recognition of this extensive list of potential biases and assumptions we argue that more research is needed to develop statistical frameworks integrating different measures of phenology and allowing to mitigate the uncertainties.

Overall, our results indicate that the growing amount of available opportunistic plant observation data provides reliable phenological information that already allows to quantify large-scale bioclimatic relationships for plant species. Opportunistic plant observations bridge the scales between dedicated individual human observation of defined phenological stages and remote-sensed phenology observations of the entire landscape. In contrast to the former, opportunistic observations are more numerous, cover more species and are collected across the entire species distribution range without requiring any additional efforts or costs. Unlike remote sensing data, opportunistic plant observations allow to collect species-specific phenology information which are urgently needed for community-level analyses and predictive phenology models. In combination with data collected within traditional phenological observation networks, these data can be used to parameterize climate and vegetation models and are expected to allow for more timely and fine-grained predictions. While we used observations without an explicit attribution to a specific pheno-phase, a logical next step would be to train image recognition models that are able to recognize different phenological stages on the available images^70^. This would enable researchers to derive estimates of different phenological stages per species and allow for even more fine-grained observations of the phenological development throughout the year.

## Methods

### Plant observation data

We selected 20 common and widespread, mostly herbaceous species with high observation counts and prominent flowering stage. Further, we considered an easy recognizability and a complete coverage of the vegetation season as essential. We also included *Sambucus nigra*, a woody species which is growing as a shrub or small tree. Similar to the other considered species it is widespread and common, has a prominent flowering stage, and its flowers and leaves are usually in a height reachable to persons using a smartphone. Observation records stem from multiple sources collecting opportunistic plant observations in Europe: the plant identification apps Flora Incognita^32^ and Pl@ntNet^71,72^; as well as the species-reporting platforms: iNaturalist^30^, Observation.org^73^, Artportalen^31^, and The Norwegian Biodiversity Information Centre (NBIC)^74^ (Fig. S1). Along the underlying selection and aggregation process we utilized dataset descriptions to make sure that observations did not follow particular observation patterns or campaigns but rather were opportunistic in nature. We pooled all observations into a single data set with 2,040,418 observation records, with both years showing a similar total observation count (943,384 in 2020 vs. 1,097,034 in 2021). Each plant observation documents the presence of a species at a certain location at a certain time. Therefore, we applied a presence-only modeling approach. Simulations have shown that phenological predictions based on presence-only observations are robust, especially when the central percentiles of the observations are determined^63^.

### Estimation of median observation date

We analyzed the median observation date as it represents a most robust phenometric for opportunistic data^63^. We rasterized Europe into 50 × 50 km grid cells and used all records per grid cell for a given species and year. For each grid cell we attempted to calculate the median date of observation as the 50th percentile based on the Weibull distribution, using the R-package phenesse^75^. Phenesse uses a parametric bootstrapping approach to calculate phenological metrics for any percentile based on the Weibull distribution. In order to reduce error and bias we conservatively chose a minimum of 25 observations per grid cell to estimate the median date per grid cell^63^. As a consequence, we calculated MOD only for grid cells that exeeded 25 observations per year (see SI Fig. S5).

Most of our observed species show a more or less symmetric observation curve with a single prominent peak representing the stage of a plant when it is most conspicuous and interesting for opportunistic observers (SI Fig. S2) - i.e. the flowering stage. This is why in most cases, the median observation date (MOD) will correspond to the peak flowering date of the plant. We divided the species into two groups: species with an median MOD in 2020 prior to DOY 172 (onset of summer) were assigned to the group “spring-flowering", while the species with a median MOD at or after that day were assigned to the group “summer-flowering" (Tab S2). We used support vector machine (SVM) regression based on elevation, latitude and longitude to interpolate the values of grid cells with too few occurrences within the area of applicability^76^. See SI Figs. S3, S5 for a comparison to the directly observed values. The difference between medians in MOD of all grid cells between both years was tested using a paired two-sided Wilcoxon Rank sum test.

### Temperature analysis

The gridded daily temperature and elevation data for Europe were retrieved from Copernicus Climate Change Service^77,78^ and re-scaled to our 50 × 50 km grid. In order to mechanistically link differences in median observation dates with local climatic conditions, we related the observed shifts per grid cell with the differences in accumulated temperature in the same area. Therefore, we used the concept of growing degree days (GDD) as a measure of thermal time (temperature accumulation above a certain baseline over time) that is commonly used in agriculture, where several phenological stages of crops are expected to fall into highly conservative ranges of GDD^79^. We calculated GDD as accumulated mean daily temperatures above a baseline of five degrees Celsius for each day of the year based on the gridded minimum and maximum temperatures consistent to previous studies predicting the flowering phenology of plants^53,54,80^.

### Effects of elevation, latitude, and longitude

Observation shifts in relation to elevation, latitude, longitude were calculated per species in both years as parameters from a multiple linear regression equation of the form:

1 $$Do{y}_{species}={m}_{elev}* elev+{m}_{lat}* lat+{m}_{lon}* lon+n+\epsilon$$

where *Doy*_*species*_ is the observed day of year per species; elev, lat, and lon are elevation, latitude, and longitude; *m*_*elev*_, *m*_*lat*_, and *m*_*lon*_ are the respective regression coefficients and *ϵ* denotes the regression error. P-values were adjusted for multiple testing according to the Bonferroni-Holm method^81^. These parameters were then used separately for elevation, latitude, and longitude to fit a quadratic regression model and to fit the relationship between median observation date to each of them (cp. Fig. 4).

### Supplementary information

Supplementary Information

## Data Availability

The dataset analysed during the current study have been deposited in the figshare repository: 10.6084/m9.figshare.23997486.
