# Supplementary material for: Opportunistic plant observations reveal spatial and temporal gradients in phenology
Source: NPJ Biodivers. 2024 Mar 6;3:5. doi: 10.1038/s44185-024-00037-7 (PMC11332049; doi:10.1038/s44185-024-00037-7)
Supplement: Supplementary file 1 — Supplementary Information [file 44185_2024_37_MOESM1_ESM.pdf]

Supplementary Table S1

Observation overview and descriptive statistics of the observed peak of observations across all grid cells where a peak of observations for the species could be determined. The difference in medians between both years was tested using a paired two-sided Wilcox Rank sum test. The median absolute deviation (MAD) reflects the variability of the observed values.

Species name and author	Total observations		Median peak observation day ± MAD		Median shift		RMSE SVM		Rsquared SVM	
	**2020**	**2021**	**2020**	**2021**	**Δ MAD**	**p-value**	**2020**	**2021**	**2020**	**2021**
Ficaria verna Huds.	35605	61940	96 ± 5.93	104 ± 10.38	8	<0.001	6.73	6.96	0.51	0.62
Lamium purpureum L.	35309	59074	100 ± 10.38	113 ± 8.90	13	<0.001	10.87	8.80	0.44	0.44
Glechoma hederacea L.	86729	118853	115 ± 8.90	125± 10.38	10	<0.001	6.08	8.61	0.74	0.57
Alliaria petiolata M.Bieb. (Cavara & Grande)	124511	92738	116 ± 8.15	130 ± 10.38	14	<0.001	4.45	7.60	0.81	0.66
Ajuga reptans L.	39348	54890	120 ± 8.90	137 ± 7.41	17	<0.001	4.89	7.26	0.74	0.63
Chelidonium majus L.	68080	88900	126 ± 11.12	138 ± 8.90	12	<0.001	10.13	11.28	0.56	0.42
Silene dioica (L.) Clairv.	48930	58358	135 ± 13.34	147 ± 7.41	12	<0.001	12.56	8.69	0.37	0.36
Veronica chamaedrys L.	46378	57108	137 ± 10.38	146 ± 5.93	9	<0.001	6.96	6.25	0.71	0.59
Aquilegia vulgaris L.	55540	58075	140 ± 10.38	152 ± 4.45	12	<0.001	5.92	4.07	0.76	0.71
Geranium robertianum L.	55679	51000	141 ± 16.31	151 ± 7.41	10	<0.001	11.20	11.61	0.55	0.43
Sambucus nigra L.	40161	38054	146 ± 13.34	163 ± 7.41	17	<0.001	12.32	15.10	0.31	0.24
Geum urbanum L.	50210	60413	153 ± 10.38	159 ± 7.41	6	<0.001	11.48	10.09	0.33	0.12
Digitalis purpurea L.	34042	32983	163 ± 14.83	172 ± 8.90	9	<0.001	7.14	7.55	0.71	0.55
Lysimachia punctata L.	25447	27956	174 ± 8.90	176 ± 5.93	2	<0.001	6.45	5.21	0.57	0.59
Centaurea cyanus L.	32223	30608	174± 9.64	175 ± 10.38	1	n.s.	8.57	7.98	0.39	0.36
Echium vulgare L.	54508	53033	174 ± 7.41	179 ± 7.41	5	<0.001	8.28	9.09	0.30	0.31
Erigeron canadensis L.	39318	47596	191 ± 10.38	197 ± 14.83	6	<0.001	11.91	16.48	0.27	0.14
Origanum vulgare L.	39576	48687	200 ± 5.93	205 ± 8.90	5	<0.001	6.29	7.34	0.30	0.23
Tanacetum vulgare L.	58392	70189	214 ± 10.38	216 ± 8.90	2	<0.01	10.75	10.70	0.24	0.37
Impatiens glandulifera Royle	45179	65964	233 ± 11.86	234 ± 7.41	1	<0.001	10.24	9.37	0.20	0.13

Supplementary Table S2

Overview of the shift in mean date of observation per species in response to 1000 m elevation and per degree latitude and longitude in the observed years. The presented values are coefficients derived from a multiple linear regression model for the originally observed (true) values per species. The adjusted R^2 is shown in the last columns and the p-values of the coefficients and significant values are marked with asterisks, where *: p<=0.05, **: p<=0.01, ***: p<=0.001, and ns: not significant. P-values were corrected for multiple testing according to the Bonferroni-Holm method. The median day of year (DOY) across all observed grid cells is shown for each species and in both years for reference.

	Flowering	Grid cells		Median DOY		Elevation		Latitude		Longitude		Adj. R^2	
**Species**	**time**	**2020**	**2021**	**2020**	**2021**	**2020**	**2021**	**2020**	**2021**	**2020**	**2021**	**2020**	**2021**
Ficaria verna	spring	234	299	96	104	20.28***	15.59***	1.98***	2.15***	0.56***	0.89***	0.52	0.62
Lamium purpureum	spring	202	274	100	113	33.80***	15.25***	4.18***	2.98***	0.77*	1.06***	0.42	0.50
Glechoma hederacea	spring	447	499	115	125	6.69***	7.50***	2.48***	2.25***	0.44***	0.49***	0.74	0.55
Alliaria petiolata	spring	477	455	116	130	9.71***	11.46***	2.08***	2.61***	0.65***	0.48***	0.81	0.66
Ajuga repta^{ns}	spring	265	292	120	137	13.26***	18.34***	2.84***	3.40***	0.11^{ns}	-0.44***	0.74	0.64
Chelidonium majus	spring	424	462	126	138	9.36***	13.64***	2.54***	2.56***	0.28*	-0.11^{ns}	0.57	0.42
Silene dioica	spring	320	327	135	147	20.35***	14.77***	2.22***	1.77***	0.70***	0.08^{ns}	0.39	0.35
Veronica chamaedrys	spring	324	338	137	146	22.56***	16.45***	2.98***	2.36***	0.20^{ns}	-0.11^{ns}	0.72	0.59
Geranium robertianum	spring	318	279	140	151	30.16***	28.25***	3.21***	3.13***	1.22***	0.77***	0.57	0.44
Aquilegia vulgaris	spring	256	251	140	152	24.23***	17.51***	2.75***	1.60***	0.79***	-0.10^{ns}	0.77	0.71
Geum urbanum	spring	268	274	153	159	15.84***	5.89^{ns}	2.28***	1.12***	0.45^{ns}	0.51**	0.33	0.17
Sambucus nigra	spring	283	260	146	163	14.83***	14.92**	2.88***	2.52***	1.05^{ns}	1.15***	0.30	0.25
Digitalis purpurea	spring	223	205	163	172	49.92***	42.69***	2.82***	1.82***	1.32***	0.18^{ns}	0.70	0.52
Centaurea cyanus	summer	210	192	174	175	32.94***	31.90***	3.65***	3.17***	-0.79*	-1.35***	0.41	0.37
Lysimachia punctata	summer	223	229	174	176	19.54***	19.82***	1.86***	1.70***	0.37*	-0.51***	0.57	0.57
Echium vulgare	summer	349	352	174	179	10.63***	13.89***	1.78***	1.88***	-0.07^{ns}	-0.17^{ns}	0.32	0.32
Erigeron canadensis	summer	225	233	191	197	10.78***	8.73 ^{ns}	2.75***	2.00***	-0.18^{ns}	0.85**	0.30	0.19
Origanum vulgare	summer	267	307	200	205	11.79***	4.23*	0.64***	-0.75***	0.07^{ns}	-0.27^{ns}	0.30	0.27
Tanacetum vulgare	summer	369	398	214	216	8.62**	6.37*	-0.89***	-1.05***	-0.11^{ns}	-0.52***	0.24	0.36
Impatiens glandulifera	summer	322	357	233	234	-5.17*	-2.80^{ns}	-1.34***	-0.94***	0.53***	0.55***	0.19	0.15

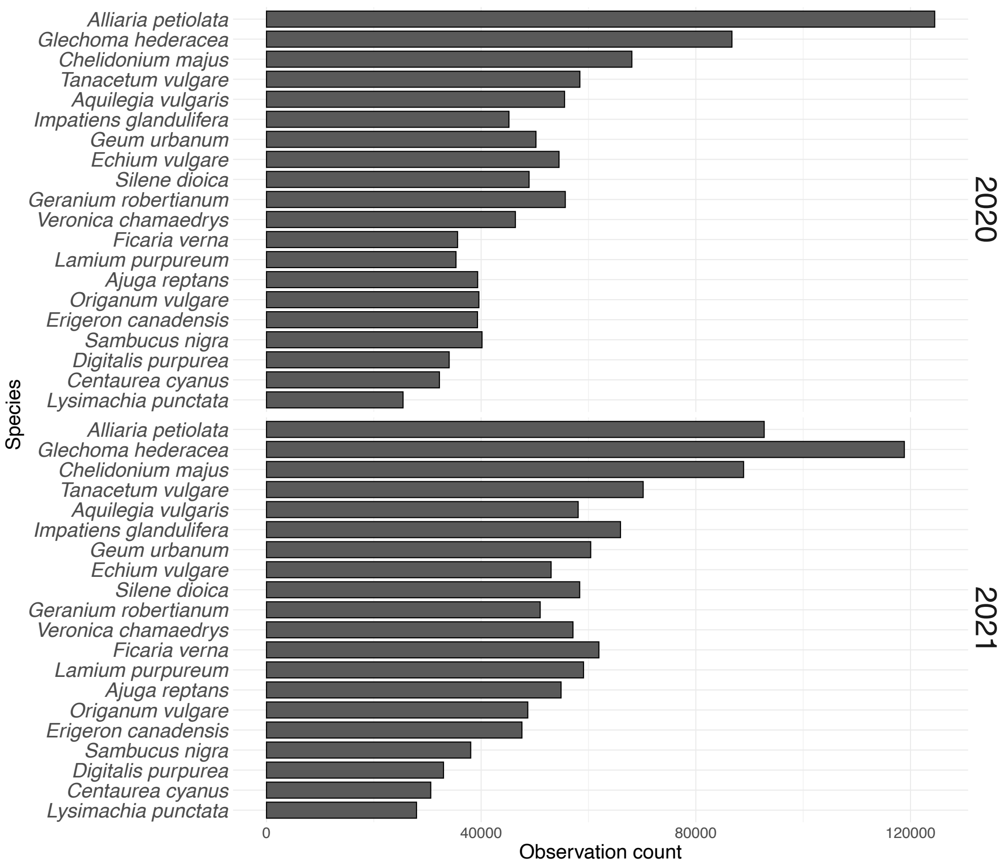

Supplementary Figure S1

Number of collected observations per species and year.

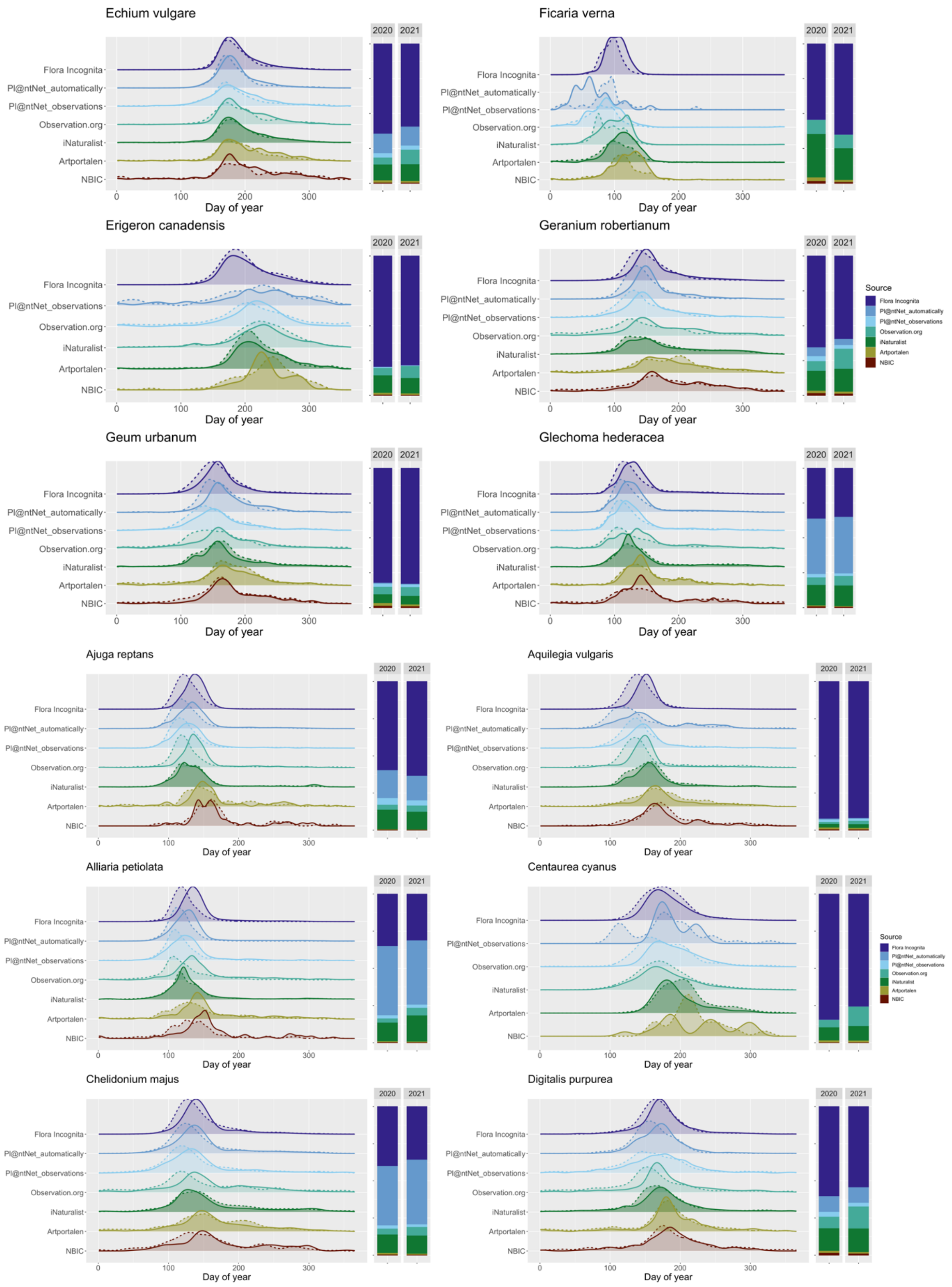

Echium vulgare
Ficaria verna
Erigeron canadensis
Geranium robertianum
Geum urbanum
Glechoma hederacea
Ajuga reptans
Aquilegia vulgaris
Alliaria petiolata
Centaurea cyanus
Chelidonium majus
Digitalis purpurea
Flora Incognita
Pl@ntNet_automatically
Pl@ntNet_observations
Observation.org
iNaturalist
Artportalen
NBIC
0
100
200
300
Day of year
2020
2021
Source
Flora Incognita
Pl@ntNet_automatically
Pl@ntNet_observations
Observation.org
iNaturalist
Artportalen
NBIC

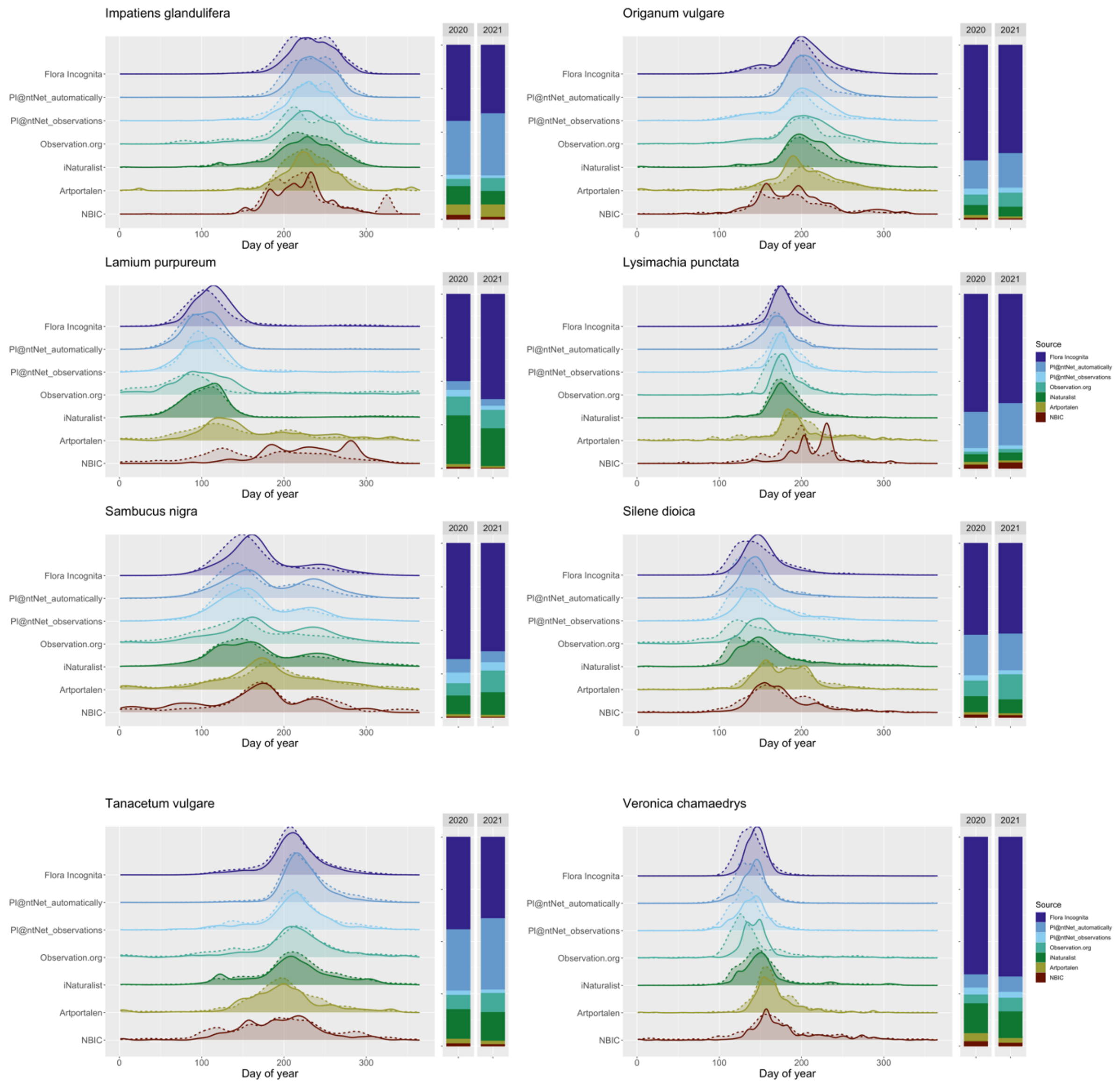

Supplementary Figure S2
Observation density curves across species and data sources. The left panels indicate the relative observation frequency throughout the year with color-coded observation sources. The two barplots on right part of the plot present the relative contribution of each source in both years to the overall number of observations.

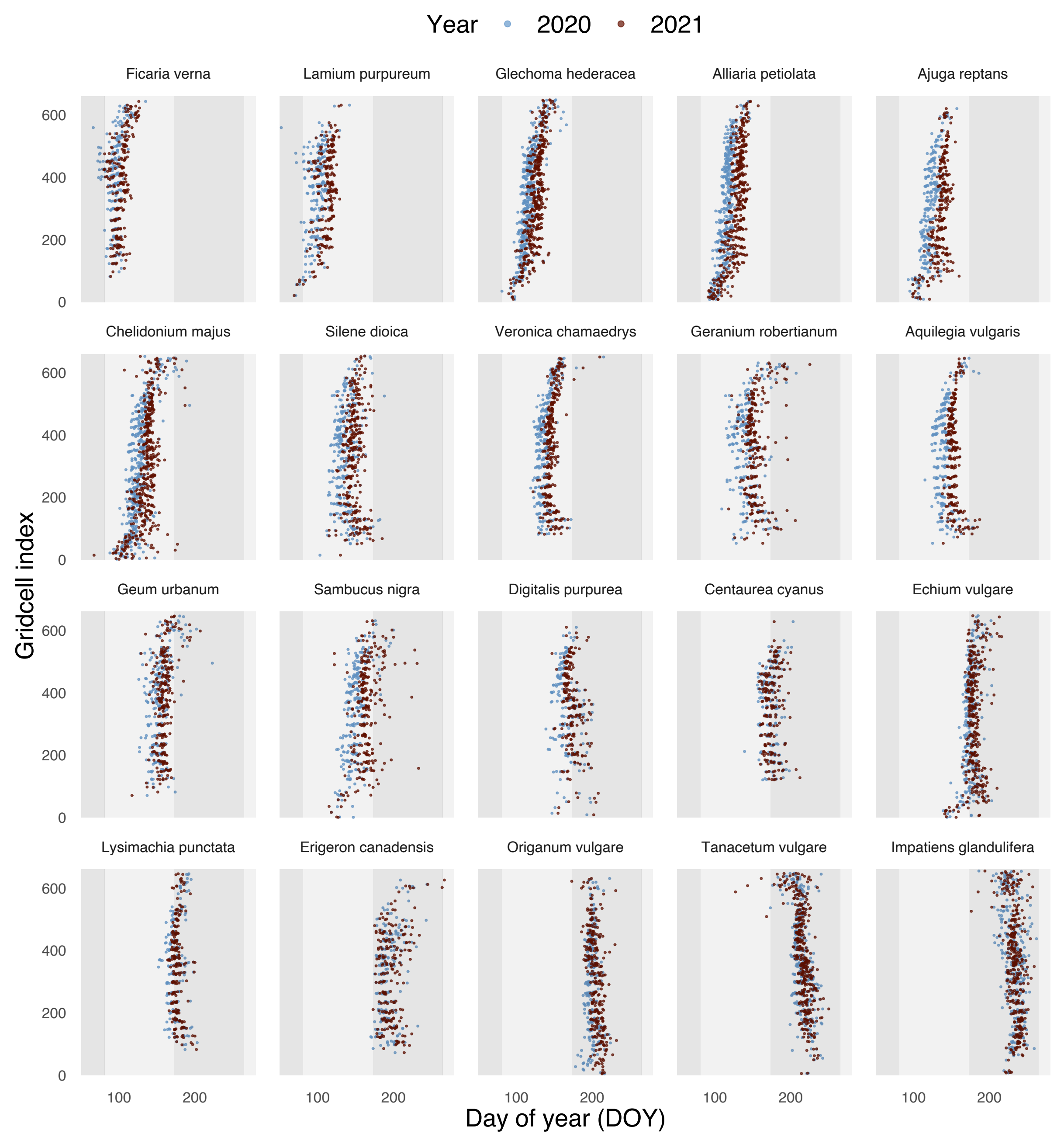

Supplementary Figure S3a

Differences in originally observed median observation (MOD) dates between 2020 (blue) and 2021 (brown) for all observed species across all gridcells where MOD could be estimated. Note that the order of the gridcells roughly corresponds to a raster pattern beginning in the south-west of Europe to the north-east. As a consequence, the gridcells with lower index numbers represent gridcells in the south while higher index numbers represent gridcells in the north. The different grey levels in the background of each panel indicate the 4 seasons in the year. Please refer to Supplementary Figure S5 for MOD in exact spatiotemporal context.

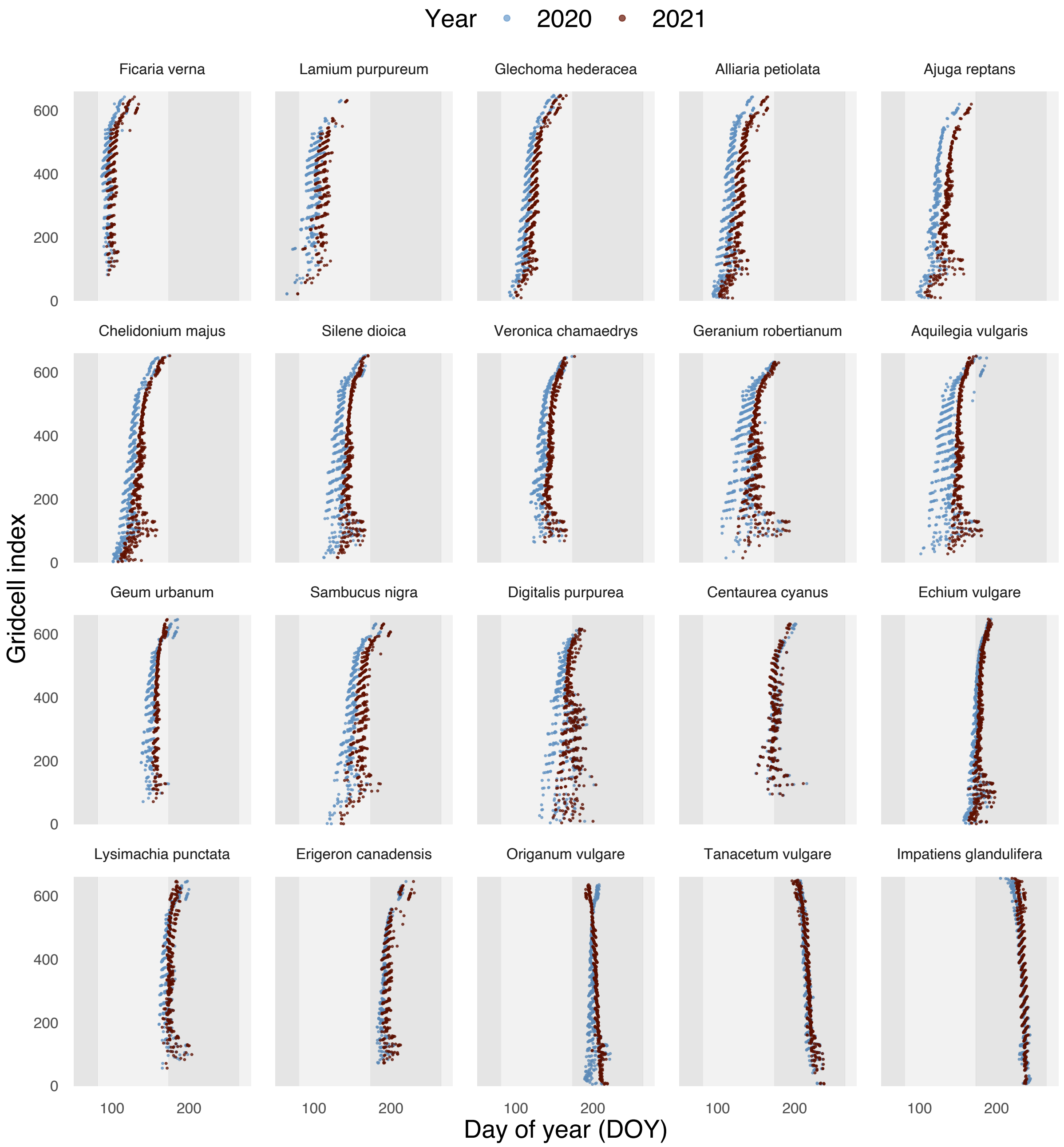

Supplementary Figure S3b

Differences in interpolated median observation (MOD) dates between 2020 (blue) and 2021 (brown) for all observed species across all gridcells where MOD could be estimated. Note that the order of the gridcells roughly corresponds to a raster pattern beginning in the south-west of Europe to the north-east. As a consequence, the gridcells with lower index numbers represent gridcells in the south while higher index numbers represent gridcells in the north. The different grey levels in the background of each panel indicate the 4 seasons in the year. Please refer to Supplementary Figure S5 for MOD in exact spatiotemporal context.

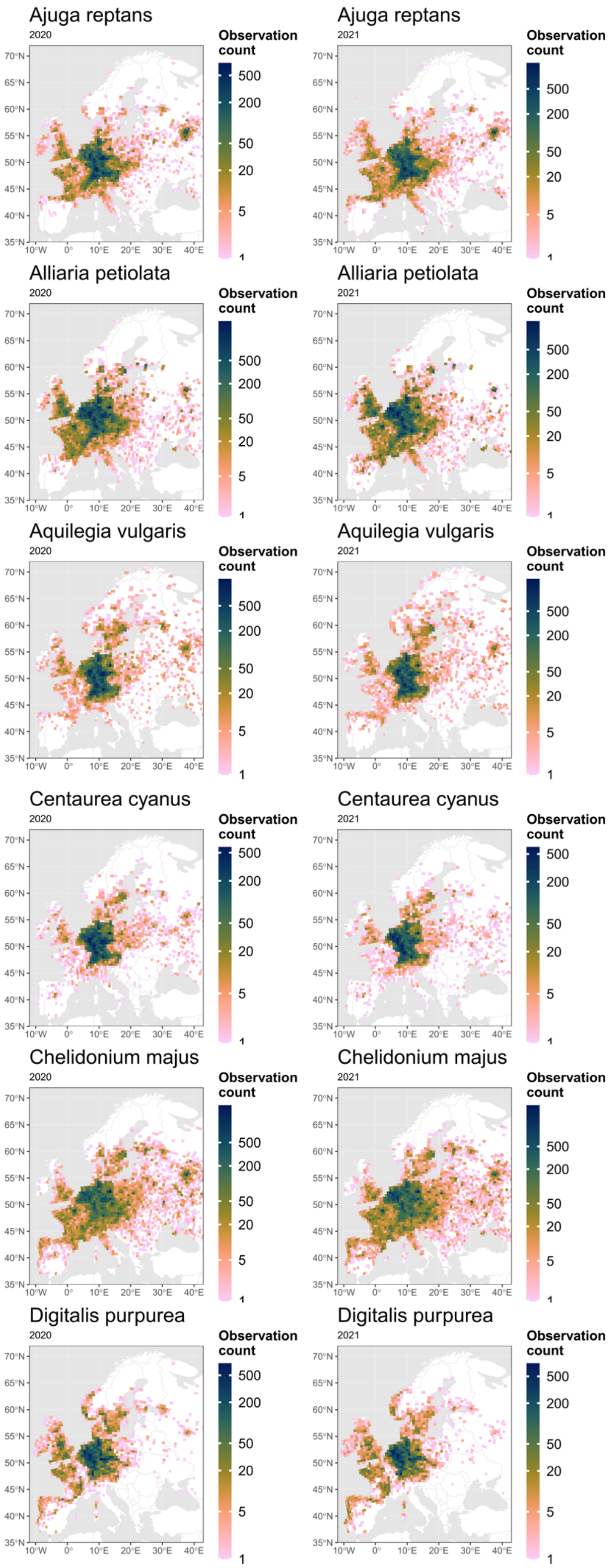

Ajuga reptans
2020
Observation count
500
200
50
20
5
1
Ajuga reptans
2021
Observation count
Alliaria petiolata
2020
Alliaria petiolata
2021
Aquilegia vulgaris
2020
Aquilegia vulgaris
2021
Centaurea cyanus
2020
Centaurea cyanus
2021
Chelidonium majus
2020
Chelidonium majus
2021
Digitalis purpurea
2020
Digitalis purpurea
2021
70°N
65°N
60°N
55°N
50°N
45°N
40°N
35°N
10°W
0°
10°E
20°E
30°E
40°E

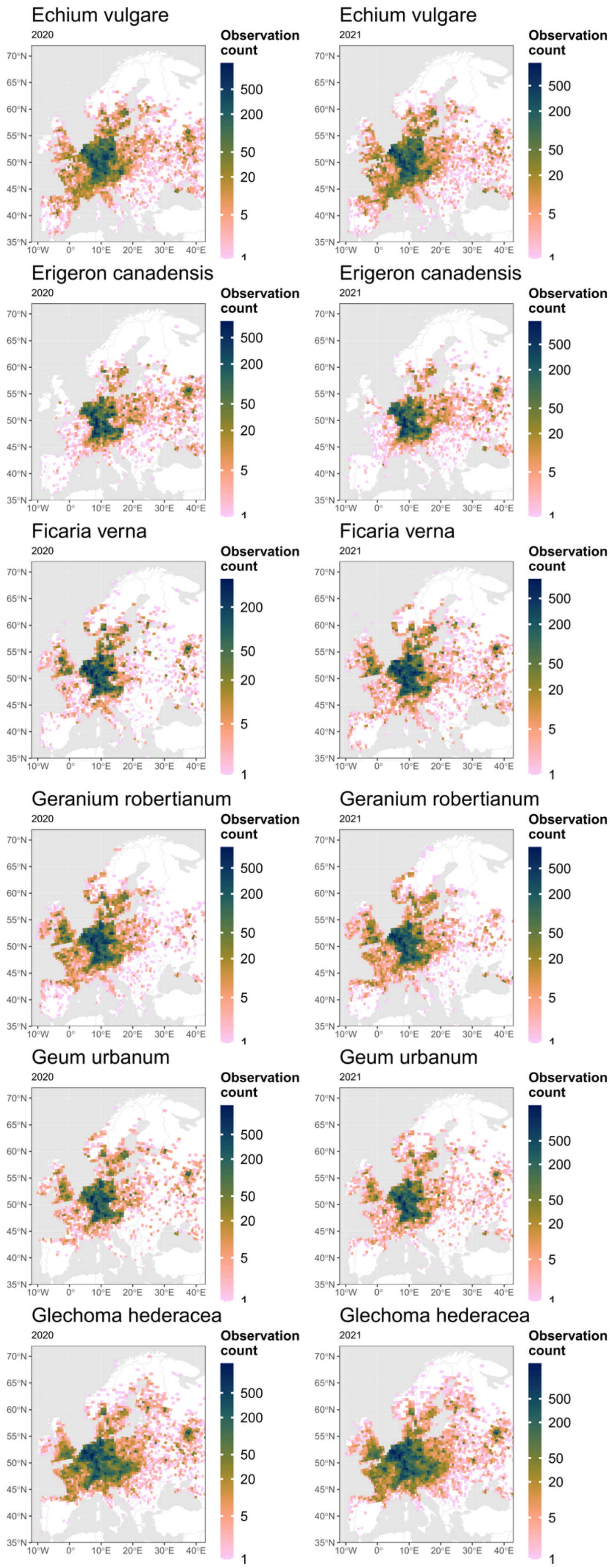
Echium vulgare
2020
Observation count
500
200
50
20
5
1
Echium vulgare
2021
Observation count
500
200
50
20
5
1
Erigeron canadensis
2020
Observation count
500
200
50
20
5
1
Erigeron canadensis
2021
Observation count
500
200
50
20
5
1
Ficaria verna
2020
Observation count
200
50
20
5
1
Ficaria verna
2021
Observation count
500
200
50
20
5
1
Geranium robertianum
2020
Observation count
500
200
50
20
5
1
Geranium robertianum
2021
Observation count
500
200
50
20
5
1
Geum urbanum
2020
Observation count
500
200
50
20
5
1
Geum urbanum
2021
Observation count
500
200
50
20
5
1
Glechoma hederacea
2020
Observation count
500
200
50
20
5
1
Glechoma hederacea
2021
Observation count
500
200
50
20
5
1
70°N
65°N
60°N
55°N
50°N
45°N
40°N
35°N
10°W
0°
10°E
20°E
30°E
40°E

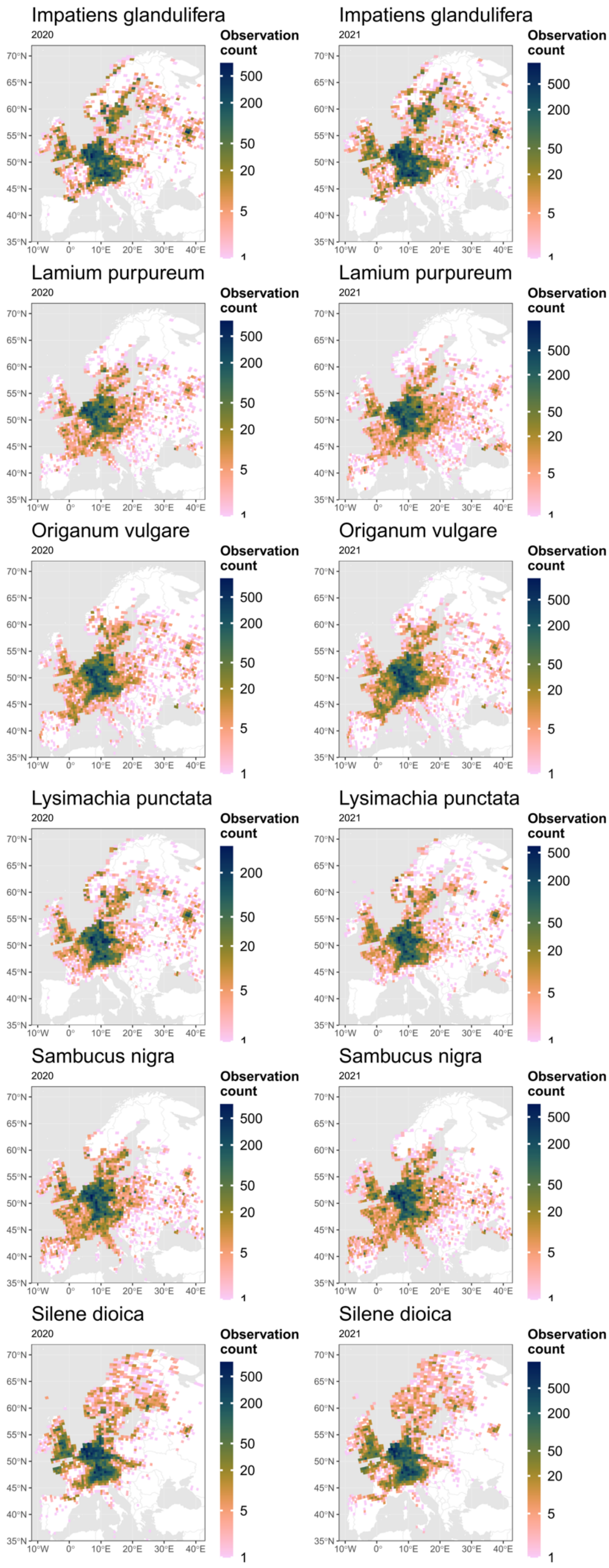

Impatiens glandulifera
2020
Observation count
500
200
50
20
5
1
Impatiens glandulifera
2021
Observation count
500
200
50
20
5
1
Lamium purpureum
2020
Observation count
500
200
50
20
5
1
Lamium purpureum
2021
Observation count
500
200
50
20
5
1
Origanum vulgare
2020
Observation count
500
200
50
20
5
1
Origanum vulgare
2021
Observation count
500
200
50
20
5
1
Lysimachia punctata
2020
Observation count
200
50
20
5
1
Lysimachia punctata
2021
Observation count
500
200
50
20
5
1
Sambucus nigra
2020
Observation count
500
200
50
20
5
1
Sambucus nigra
2021
Observation count
500
200
50
20
5
1
Silene dioica
2020
Observation count
500
200
50
20
5
1
Silene dioica
2021
Observation count
500
200
50
20
5
1
70°N
65°N
60°N
55°N
50°N
45°N
40°N
35°N
10°W
0°
10°E
20°E
30°E
40°E

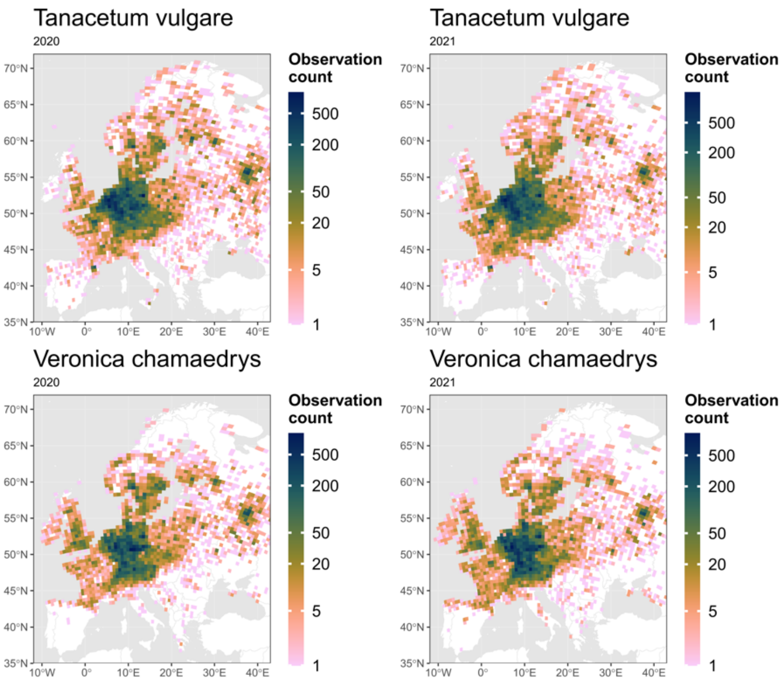

Supplementary Figure S4

Overview of observation count per gridcell for all considered species and years in comparison. Note that the color coding is on a log-scale.

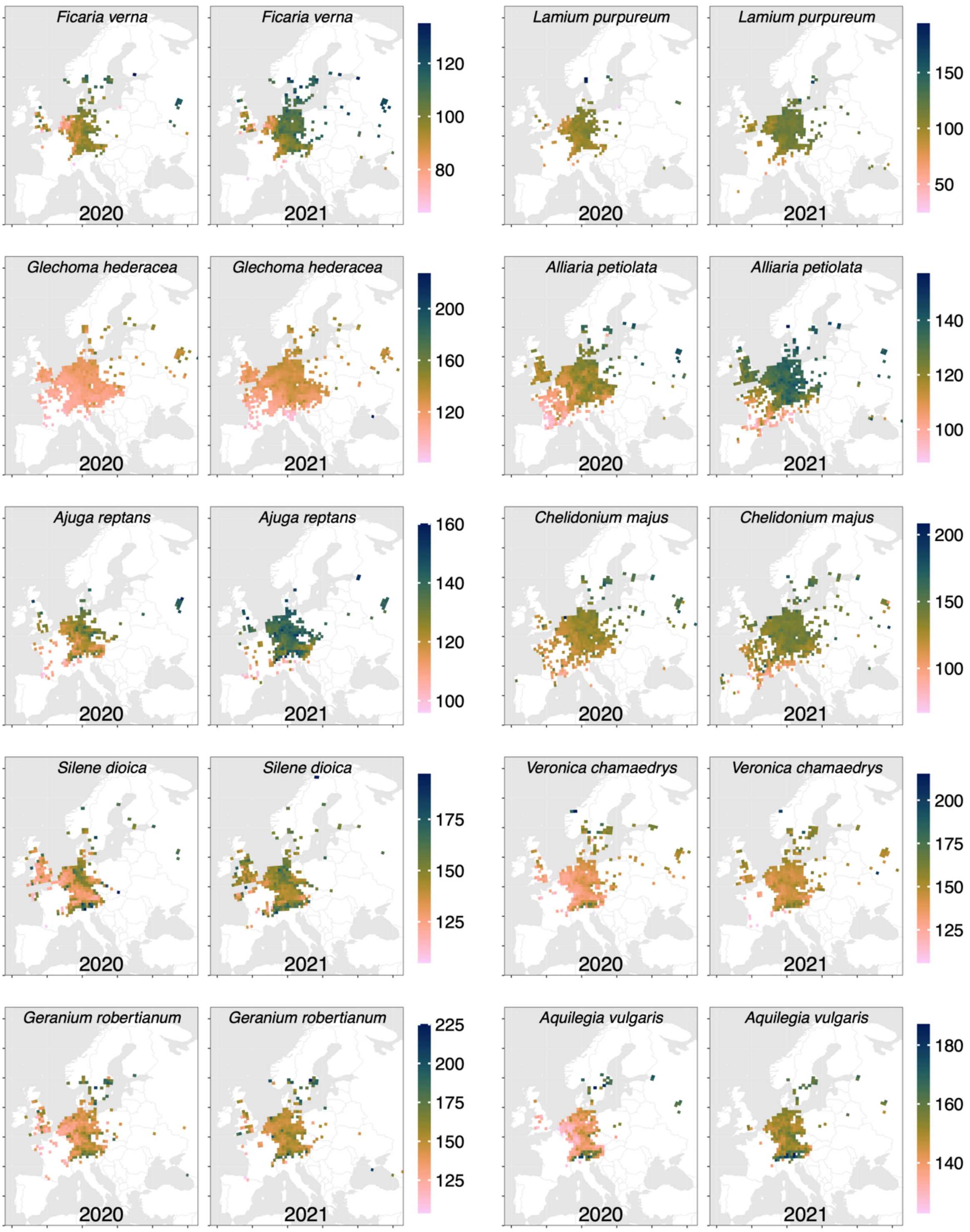

Supplementary Figure S5a

Median date of observation (MOD) per grid cell for the ten earlier flowering species for both years in comparison. Note that the scales are unique for each species, comprising the total range of observed values across both years. Therefore, colors are only comparable within species.

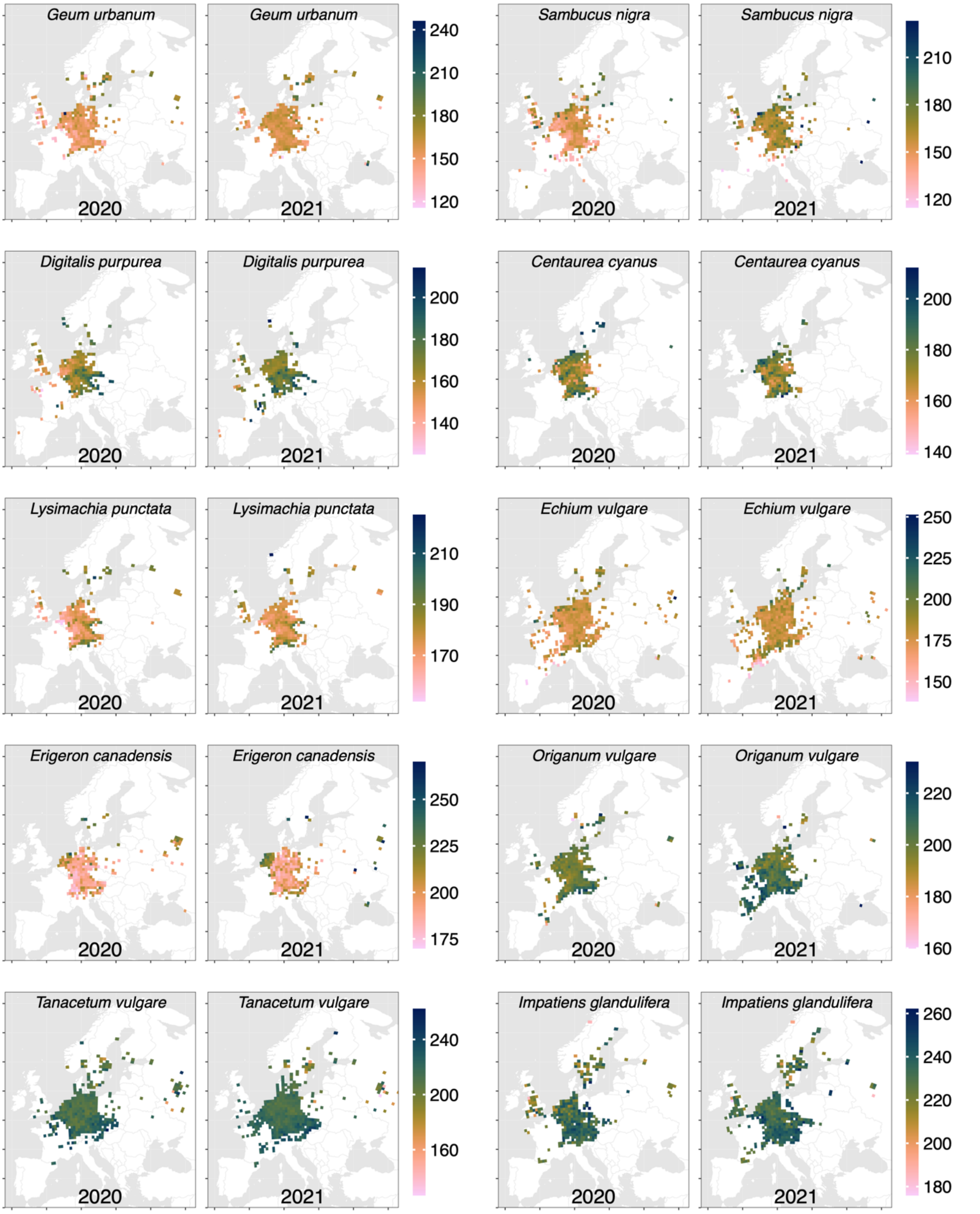

Supplementary Figure S5b

Median observation date (MOD) per grid cell for the ten later flowering species for both years in comparison. Note that the scales are unique for each species, comprising the total range of observed values across both years. Therefore, colors are only comparable within species.

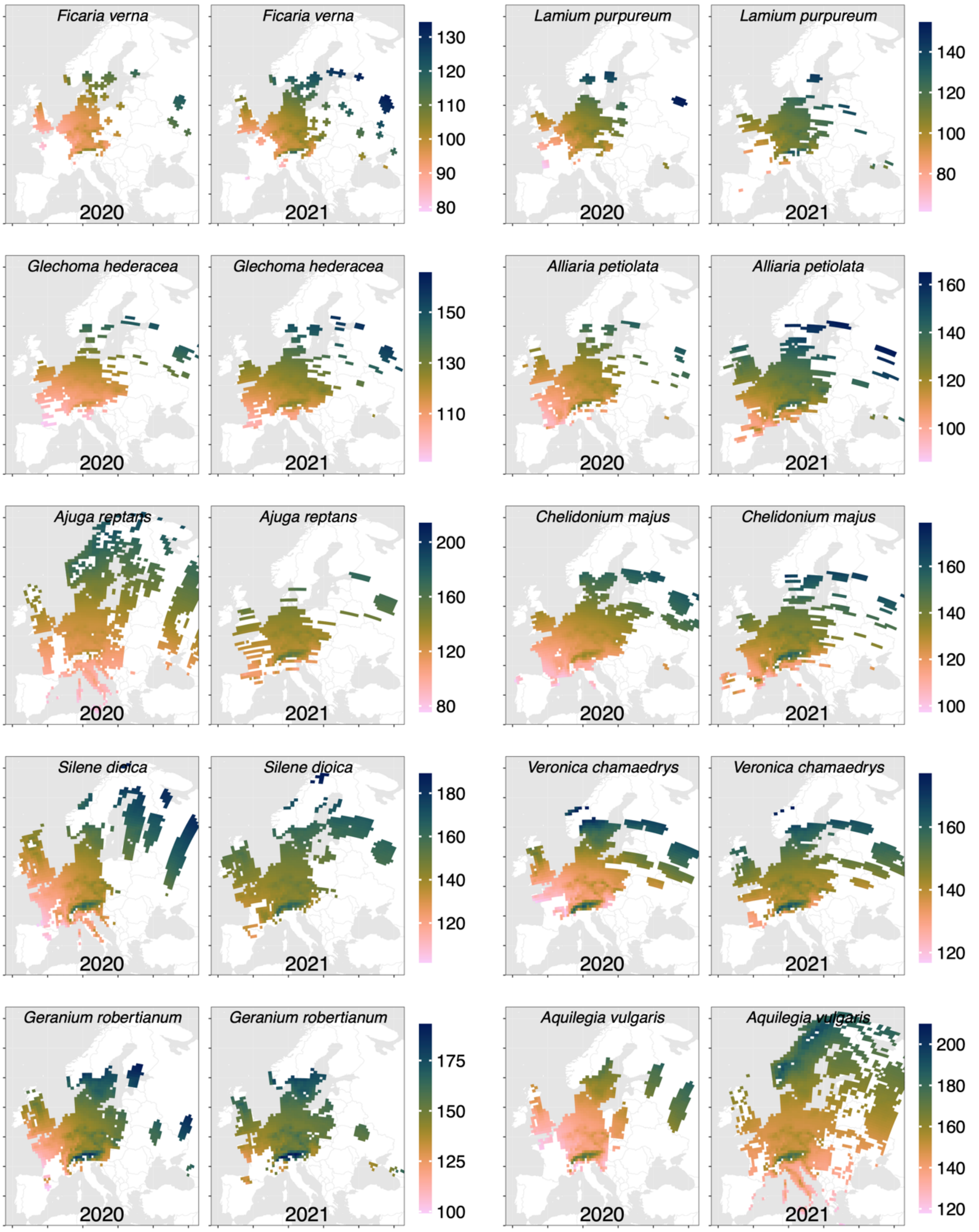

Supplementary Figure S6a

Interpolated median observation date (MOD) per grid cell for the ten earlier flowering species for both years in comparison. Note that the scales are unique for each species, comprising the total range of observed values across both years. Therefore, colors are only comparable within species.

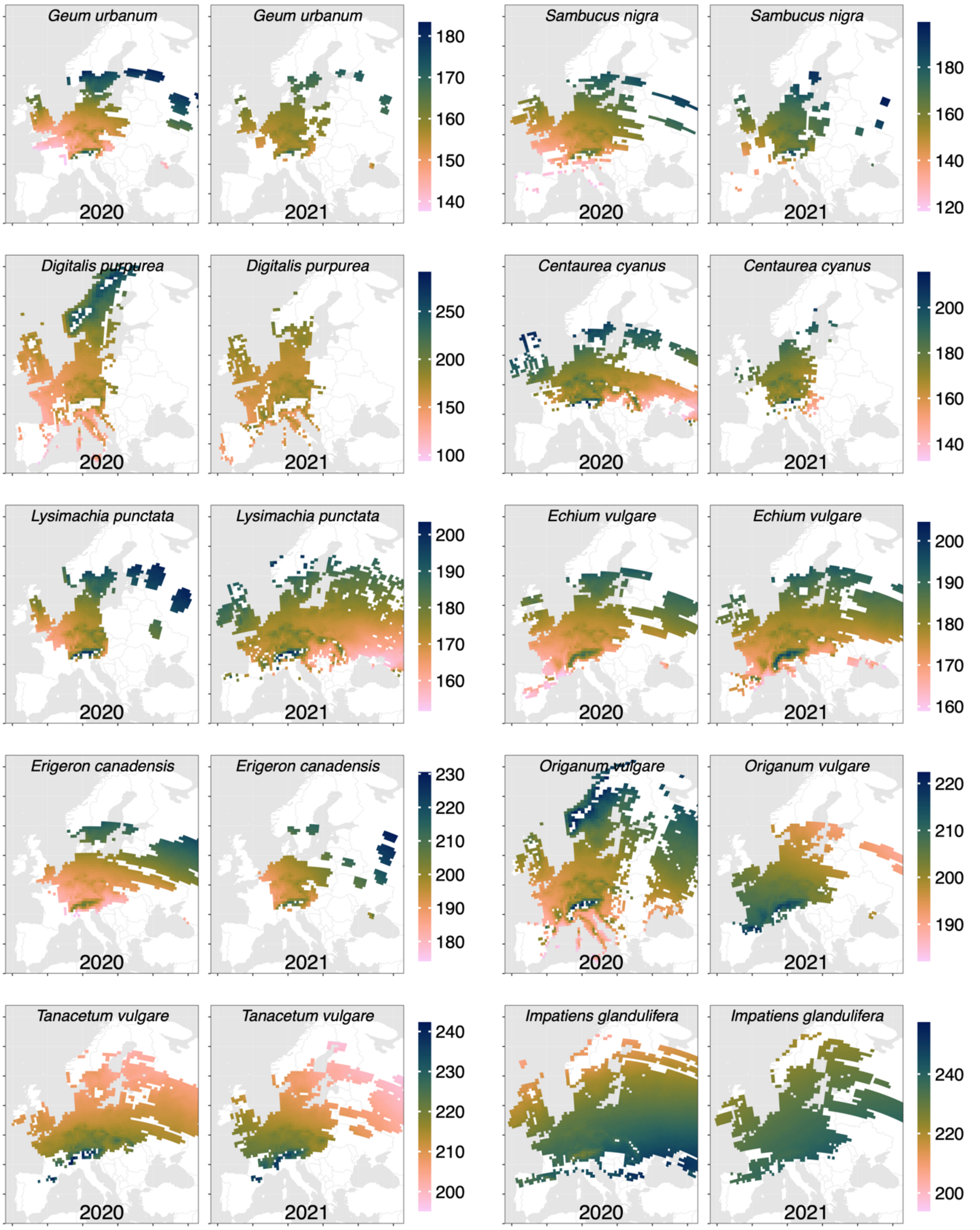

Supplementary Figure S6b

Interpolated median date (MOD) per grid cell for the ten later flowering species for both years in comparison. Note that the scales are unique for each species, comprising the total range of observed values across both years. Therefore, colors are only comparable within species.

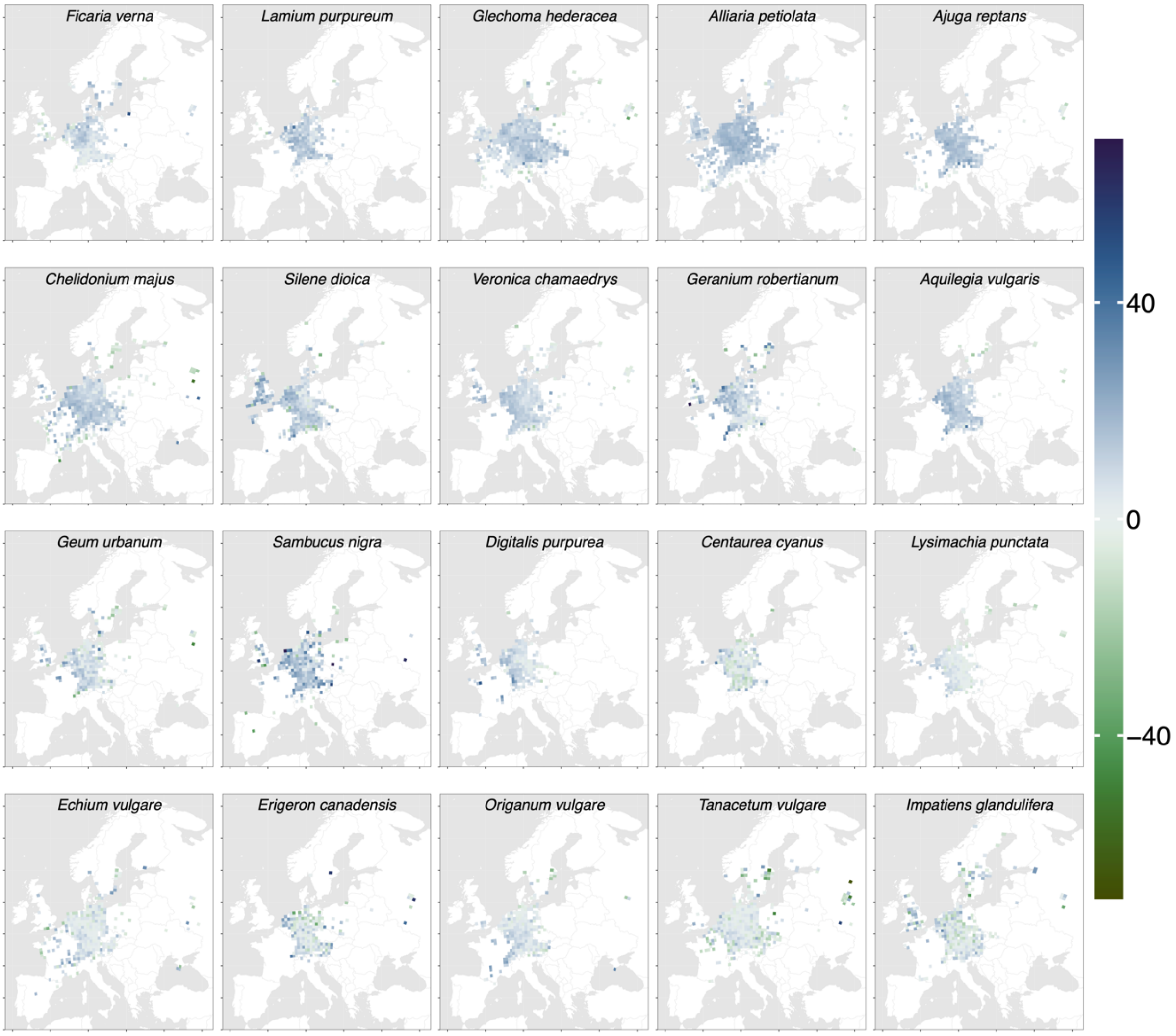

Supplementary Figure S7

Change of median observation date (MOD) in 2021 relative to 2020 based on the observed values for grid cells with observations from both years.

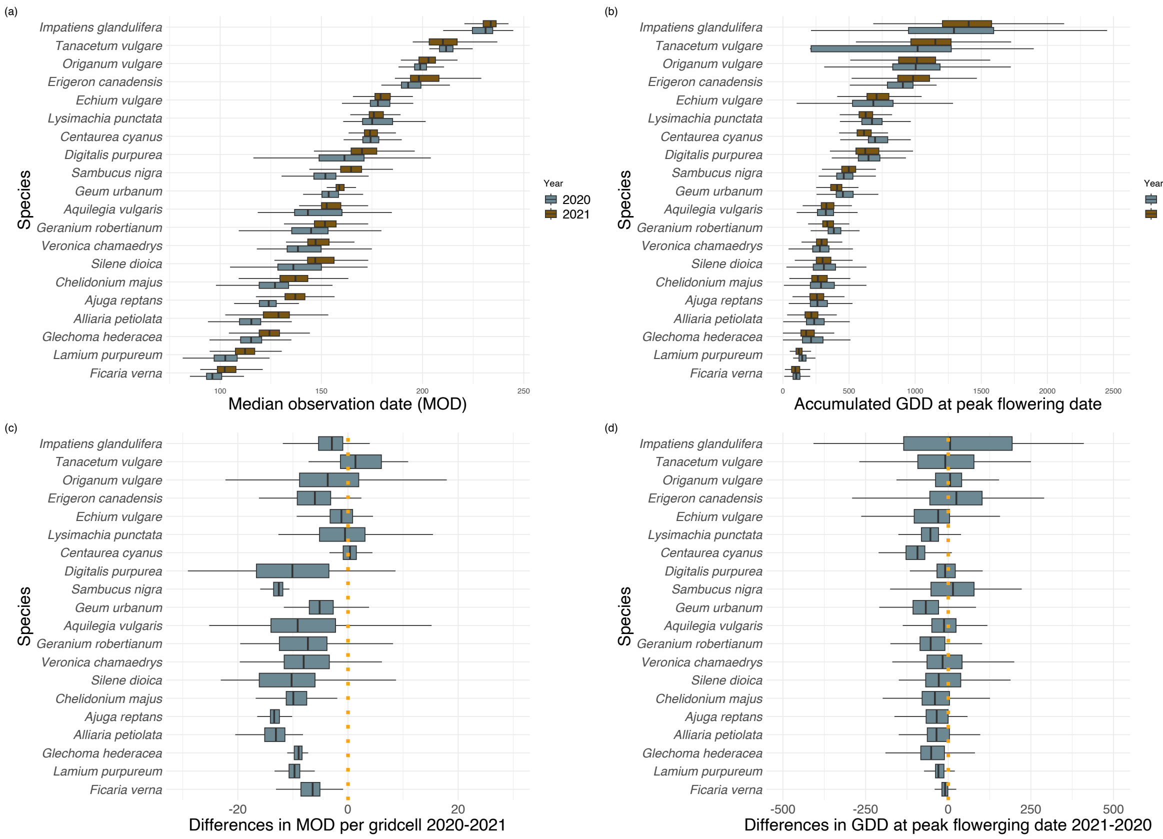

Supplementary Figure S8

The boxplots represent the distribution of species observations in all gridcells. (a) represents the temporal distribution of median observation dates (MOD) for all observed species in both observed years. (b) shows the accumulated temperature above the baseline of 5°C (GDD) in each gridcell at the time when peak flowering of this species was observed. (c) shows the distribution of differences for MOD between the years for all observed species. (d) shows the distribution of differences between the years for accumulated temperature (GDD) per gridcell for all observed species.
